# Safety and tolerability of *Bifidobacterium longum* subspecies *infantis* EVC001 supplementation in healthy term breastfed infants: a phase I clinical trial

**DOI:** 10.1186/s12887-017-0886-9

**Published:** 2017-05-30

**Authors:** Jennifer T. Smilowitz, Jackelyn Moya, Melissa A. Breck, Chelsea Cook, Annette Fineberg, Kathleen Angkustsiri, Mark A. Underwood

**Affiliations:** 1 Department of Food Science and Technology, University of California, Davis, CA USA; 2 Foods for Health Institute, University of California, One Shields Ave, Davis, CA 95616 USA; 3 Sutter Health, Sutter Davis Hospital, Davis, CA USA; 4 UC Davis MIND Institute, University of California, Davis, CA USA; 5 Department of Pediatrics, UC Davis Children’s Hospital, Sacramento, CA USA

**Keywords:** *Bifidobacterium longum* subspecies *infantis*, Breast milk, Gut microbiome, Human milk oligosaccharides, Infant, Probiotics, Supplementation, Tolerability

## Abstract

**Background:**

Historically, bifidobacteria were the dominant intestinal bacteria in breastfed infants. Still abundant in infants in developing nations, levels of intestinal bifidobacteria are low among infants in developed nations. Recent studies have described an intimate relationship between human milk and a specific subspecies of *Bifidobacterium, B. longum* subsp. *infantis* (*B. infantis*), yet supplementation of breastfed, healthy, term infants with this organism, has not been reported. The IMPRINT Study, a Phase I clinical trial, was initiated to determine the safety and tolerability of supplementing breastfed infants with *B. infantis* (EVC001).

**Methods:**

Eighty mother-infant dyads were enrolled in either lactation support plus *B. infantis* supplementation (BiLS) or lactation support alone (LS). Starting with Day 7 postnatal, BiLS infants were fed 1.8–2.8 × 10^10^ CFU *B. infantis* EVC001 daily in breast milk for 21 days. Mothers collected fecal samples, filled out health questionnaires, and kept daily logs about their infants’ feeding and gastrointestinal symptoms from birth until Day 61 postnatal. Safety and tolerability were determined from maternal reports.

**Results:**

There were no differences in the mean gestational age at birth, weight 1 and 2 months postnatal, and breast milk intake between groups. The mean Log_10_ change in fecal *Bifidobacterium* from Day 6 to Day 28 was higher (*p* = 0.0002) for BiLS (6.6 ± 2.8 SD) than for LS infants (3.5 ± 3.5 SD). Daily stool number was higher (*p* < 0.005) for LS and lower (*p* < 0.05) for BiLS infants during supplementation than at Baseline. During supplementation, watery stools decreased and soft stools increased by 36% over baseline in BiLS infants *(p* < 0.05) with no significant changes in stool consistency for the LS infants. None of the safety and tolerability endpoints, including flatulence, bloody stool, body temperature, ratings of gastrointestinal symptoms, use of antibiotics or gas-relieving medications, infant colic, jaundice, number of illnesses, sick doctor visits, or diagnoses of eczema were different for the groups at any point.

**Conclusions:**

The *B. infantis* EVC001 supplement was safely consumed and well-tolerated. Stools were fewer and better formed in infants in the BiLS group compared with LS group. Adverse events were those expected in healthy infants and not different between groups.

**Trial registration:**

ClinicalTrials.gov NCT02457338. Registered May 27, 2015.

**Electronic supplementary material:**

The online version of this article (doi:10.1186/s12887-017-0886-9) contains supplementary material, which is available to authorized users.

## Background

Breast milk not only provides nutrition but has evolved to protect and support development of the vulnerable infant. Breast milk delivers a wide spectrum of biologically active molecules that aid in the development and maturation of the gut, and the innate and acquired immune systems, and support the growth of protective intestinal microbiota. Advances in mass spectrometry have revealed detailed chemical structures of the complex and diverse free and conjugated glycans in human milk [1]. Specifically, human milk oligosaccharides (HMO), the third most abundant component in human milk (~10–20 g/L) [2, 3], are a group of complex sugars that are non-digestible by the human infant and support the competitive growth of protective bifidobacterial strains within the intestine [4]. Bifidobacteria were first identified in the feces of breastfed infants by Henry Tissier at the Pasteur Institute in 1900 [5]. Recent research has demonstrated that the gut of the breastfed infant is dominated by strains of *Bifidobacterium* until cessation of breastfeeding [6, 7].

Recent breakthroughs in microbiology have led to a detailed description of the natural colonization of a protective subspecies of *Bifidobacterium, (B. infantis)* in breastfed infants, and the role of human milk in delivering complex HMO as natural prebiotics to selectively enrich the growth and function of *B. infantis*. Unlike other bifidobacterial strains, *B. infantis* is unique in its ability to consume HMO as its sole source of carbon through specific solute binding proteins, transporters and glycosidic hydrolases that are encoded in its genome [8–14]. The subsequent effects of HMO metabolism by *B. infantis* include its production of acetate and lactate; its direct binding to intestinal cells; and its stimulation of anti-inflammatory and inhibition of pro-inflammatory cytokines by intestinal cells [15–18]. In animal models of necretozing enterocolitis (NEC), *B. infantis* supplementation was found to attenuate the inflammation [19] and increase intestinal permeability [20] associated with NEC.

The dominance of fecal *Bifidobacterium* and *B. infantis* has declined over recent decades in developed countries [21, 22]. For example, in a U.S. cohort of exclusively breastfed infants, 30% of the total gut microbiome was represented by the genus *Bifidobacterium,* whereas only 13% of total intestinal bifidobacterial populations was represented by the subspecies *B. infantis*. On the other hand, in a trial conducted in Bangladeshi breastfed term infants, 77% of the total gut microbiome was represented by the genus *Bifidobacterium* and 57% was represented by *B. infantis* [21].

Differences in postnatal intestinal microbial colonization may explain in part the higher incidence of immune-mediated diseases, such as allergy and asthma, in children born by cesarean section compared with those born vaginally [23–27], and the increase in type 1 diabetes and food allergies in children in developed countries [28]. Thus, the early colonization and establishment of a healthy microbiome in infancy is critical for establishing life-long health.

One of the main objectives of the Infant Microbiota Probiotic Intake (IMPRINT) Study was to determine the safety and tolerability of supplementing breastfed, term infants with *B. infantis* EVC001. Although *B. infantis* was reported to be well-tolerated when provided to premature infants, such data have not yet been reported for healthy term infants [29]. In this Phase I clinical trial, safety and tolerability were determined by measuring the changes in infant weight, parental reports of gastrointestinal (GI) symptoms, illnesses, use of antibiotics or gas-relieving medications, and sick doctor visits throughout the study duration. Additional outcome measures related to infant fecal microbial composition were prespecified in the study protocol and will be reported elsewhere.

## Methods

### Study population

Between January 2015 and April 2016, healthy women who were pregnant or who had recently delivered healthy term infants and lived within Yolo and Sacramento counties in California were recruited and subsequently provided written informed consent to enroll in the study. Enrollment criteria for study participation were based on limiting the number of confounding variables that could influence the infant gut microbiome. Inclusion and exclusion criteria for mothers were as follows: women 21 to 45 years of age, in their third trimester of pregnancy or had delivered an infant within the past 4 days, planned to exclusively breastfeed their infants for at least the first 3 months postnatal, lived in a developed nation for the past 10 years, did not plan to administer probiotic supplements to their infants during the study duration unless they were allocated to the *B. infantis* group, who had not been diagnosed with any chronic metabolic disease or obesity, and who were non-smoking. Medications during labor, including antibiotics, were recorded but not used as an exclusion criterion. Inclusion and exclusion criteria for infants born to qualified mothers included gestational age at birth ≥37 weeks, birth without medical complications (such as respiratory distress syndrome, birth defects, and infection), no exposure to any oral or intravenous antibiotics 72 h postnatal, and no consumption of infant formula 24 h prior to the Day 7 postnatal at-home lactation consultation visit.

### Study design

This IMPRINT study was a parallel, partially-randomized, controlled 2-month trial. The University of California Davis Institutional Review Board approved all aspects of the study (IRB #: 631,099). This trial was registered on ClinicalTrials.gov Identifier: NCT02457338. Prior to the initiation of the study, three separate randomization schemes were generated using a random number generator in Excel. Participants were stratified to one of the three randomization schemes based on mode of delivery—vaginal delivery, cesarean section (time of membrane rupture before delivery ≤6 h), or cesarean section (time of membrane rupture before delivery >6 h). Stratified randomization was utilized because mode of delivery, as well as the time when membranes rupture before cesarean section delivery, have been shown to influence early infant intestinal microbial colonization [30]. Randomization to lactation support alone (LS) or lactation support plus *B. infantis* supplementation (BiLS) was in a 1:1 ratio for all three randomization schemes in blocks of ten. After receiving informed consent for the infant, the clinical coordinator assigned enrollment identification numbers according to the schedule. The first fifteen participants enrolled in the study were not included in the stratified randomization schedule due to unavailability of the *B. infantis* product during the trial period. The first eight infants enrolled in the study were assigned to the LS group, but three had withdrawn or were screen-failed and thus only five infants received the intervention. The subsequent seven infants were assigned to the BiLS group, but three had withdrawn or were screen-failed and thus only four infants received the intervention. Parity and mode of delivery were not different between the two assigned groups for these fifteen participants.

After meeting major postpartum study criteria at enrollment (Day 3 or 4), infants were randomized into the BiLS or LS group. On Day 7, infants were screened for the consumption of infant formula within the past 24 h. On Days 3 or 4, 7, 15, 22, 33, and 61, study personnel visited mothers’ homes to conduct study procedures. On all six visits, mothers filled out questionnaires about their and their infants’ health, GI symptoms, occurrence of fever, illness, and number and reasons for sick doctor visits. Mothers collected infant stool samples from their infants’ diapers before Day 6 (baseline) and on Days 10, 14, 21, 25, 29, 32, 40, 50, and 60 and stored them in their kitchen freezers. Infant weight was measured by study personnel with a digital infant scale (Tanita) on Days 33 and 61. Participants received breastfeeding support at their homes by the study’s internationally board certified lactation consultant (IBCLC) prenatally and on Days 3 or 4, 7, and 15. On Days 22, 33, and 61 postnatal, study personnel transported samples from participants’ homes to the UC Davis campus on dry ice and stored at −80 °C.

Infants randomized into the BiLS group received one daily serving of *B. infantis* in their homes for 21 consecutive days starting on Day 7 and continuing through Day 27. During the Day 7 lactation consultation visit, mothers were trained by their lactation consultant to mix each *B. infantis* serving with 5 mL of their breast milk in a plastic medicine cup, and to syringe or finger-feed the mixture to their infants. Each daily serving of *B. infantis* EVC001 (ATCC accession Number SD-7035) consisted of one 625-mg sachet, delivering a minimum 156 mg of live bacteria (minimum 1.8 × 10^10^ CFU) plus 469 mg of lactose as the excipient. The 18 billion CFU per sachet was the minimum guaranteed CFU count as determined by the product specification. Because this is a live microorganism there is potential loss over time. As such, the sachets were produced with a 50% overage to account for potential losses during packaging and long term storage. This means the range of the dose delivered was 18–28 billion CFU per dose. The product was stored in the freezer at −20 °C and suffered no loss from the first infant enrolled to the last infant enrolled. Mothers received 21 sachets, plus four extra sachets that were to be used in the event of damage or misplacement. All sachets were kept frozen in mothers’ kitchen freezers until time of use, and mothers were instructed to keep all used and unused sachets provided. Compliance was assessed on Days 22 and 33 by counting and recording the number of empty *B. infantis* sachets.

Infant stool samples without labeled group assignments were provided to Evolve BioSystems, Inc. (Davis, CA USA) for the analysis of total infant fecal *Bifidobacterium*. Group assignments were unblinded to Evolve BioSystems, Inc. post microbial analysis. For DNA extraction, approximately 100 mg of the frozen collected stool samples were extracted using the Zymo Research Fecal DNA kit, according to the manufacturer’s instructions. Polymerase chain reaction (PCR) amplification was conducted using methods as previously described, with minor modifications [31]. Briefly, 5 μL of extracted DNA were used as template for a 20-μL reaction, using primers Bif F (5′-GCGTGCTTAACACATGCAAGTC-3′), Bif R (5′- CACCCGTTTCCAGGAGCTATT-3′), and Bif P (5′-TCACGCATTACTCACCCGTTCGCC-3′). Reactions were carried out with Taqman Universal MasterMix II with Uracil-N glycosylase (Life Technologies) [31] using a Life Technologies QuantStudio 3 Real-Time PCR machine. Samples were assayed in duplicate. A standard curve was prepared from *Bifidobacterium longum* subsp. *infantis E*VC001 using the same extraction protocol as used for the stool samples for quantification of fecal *Bifidobacterium*.

### Infant gastrointestinal health and tolerability

Infant GI tolerability during *B. infantis* EVC001 supplementation was assessed by mothers on a daily basis starting with Day 1 (or retrospectively if mothers were enrolled on Day 4 postnatal) until Day 61. On each day, mothers recorded the following information about their infants in daily logs: consumption of breast milk defined as suckling at the breast for at least five minutes or consuming any volume in a bottle; intake of other liquids or solids; amount of infant formula consumed; intake of probiotics that were not used in the study; intake of any oral antibiotics or administration of intravenous antibiotics; intake of any over-the-counter or prescribed medications; intake of vitamins, supplements, and herbs; number of spit-ups—less than five, five to ten, or more than ten; number of stools; consistency of stools using a modified Amsterdam infant stool scale—watery, soft, formed, hard [32] (Additional file 1); blood in the stool; body temperature above 100.3 °F; ratings of GI-related symptoms using a continuous scale of 0 (“not noticeable”) to 10 (“most severe”), including general irritability (“how irritable was your baby?”), upset (“if your baby vomited or spit up, how upset was he/she after?”), and discomfort (“rate your baby’s discomfort in passing stool or gas”). Mothers also rated the frequency of their infant’s flatulence as “never, sometimes, often, very often” on a daily basis. Mothers filled out questionnaires to report any adverse events experienced by their infants during each at-home visit (Days 7, 15, 22, 33 and 61). Mothers recorded the following about their infants on weekly questionnaires: episodes of colic—defined as crying for more than 3 h per day for at least 3 days per week [33], eczema diagnosis by a primary-care provider, number of sick doctor visits, illnesses, and medications used (Additional file 2).

### Statistics

Data from the daily logs and retrospective questionnaires were binned into three time periods: baseline (Days 1–6), intervention (Days 7–27), and post-intervention (Days 28–61). For retrospective questionnaires, Day 7 data were binned as baseline. Means and proportions were calculated for continuous variables and categorical variables across all three time periods. Proportions for binary categorical variables were calculated as number of days reported/total number of days in each study period, and number of infants/total number of infants in each intervention group. The calculated values were multiplied by 100 to generate percentages.

For this Phase I study, the sample size was based on differences in infant fecal *B. infantis*, which was calculated using the means and standard deviations from a previous study on breastfed infants [22]. To detect a standardized inter-group difference of 1.3 z-scores in infant fecal *B. infantis* with 90% power and α = 0.05, assuming equal standard deviations with a 20% attrition rate, 30 infants were needed in each group. Intent-to-treat analysis was performed of mother-infant dyads who initiated the study by Day 7 when final screening criteria were met. Statistical analyses were performed in IBM SPSS Statistics version 24 and figures were generated in PRISM v.7. Statistical significance was considered as *p* < 0.05. Continuous data were checked visually for normality with histograms and quintile-quintile plots as well as numerically with the Shapiro-Wilk test and equality of variances using Levene’s statistic. Non-normal data were Log_10_ transformed and confirmed again for normality prior to conducting parametric analyses.

To determine differences in total infant fecal *Bifidobacterium* between groups, Mann-Whitney U test was conducted using GraphPad Prism v7. Baseline demographics, maternal health, pregnancy history, and infant feeding and GI symptoms were compared between the LS and BiLS groups using the Pearson Chi-square Test for Independence (categorical variables), Mann-Whitney *U* Test, or one-way ANOVA (continuous variables). For normally-distributed continuous data, repeated measures ANOVA was performed with group and time as fixed factors, parity as the covariate, and group by time as the interaction term. If time was significant, multiple comparison post-hoc analysis with Bonferroni correction was carried out to compare baseline, intervention, and post-intervention data. Group differences in stool consistency, flatulence, and spitting-up were analyzed by logistic regression.

## Results

### Study participation

**Fig. 1:**
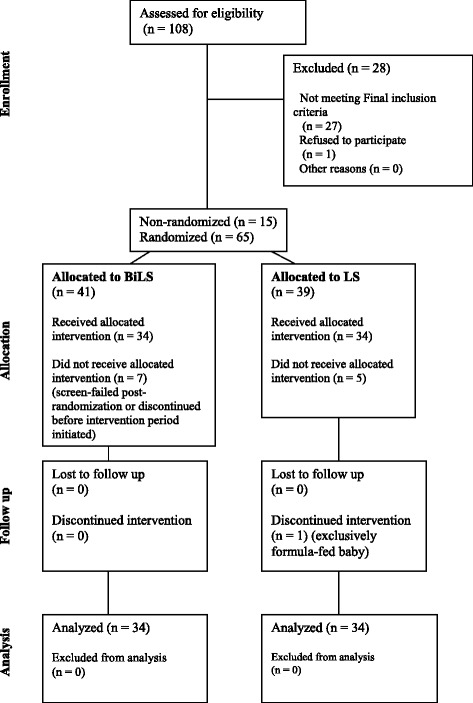
Consort diagram. Consort diagram describing the number of participating mothers who were screened, randomized into the intervention groups, screened-failed post-randomization, and withdrew throughout the study period

One-hundred and eight mothers were screened for eligibility to participate in the study. Eighty women met initial study criteria, of which fifteen were non-randomly assigned and sixty-five were randomly assigned into the LS (*n* = 39) and BiLS *(n* = 41) groups (Fig. 1). Screen failures were due to the use of infant formula within 24 h of the Day 7 lactation consultation visit. Mothers withdrew from study participation for feeling overwhelmed with a new infant and/or unexpectedly discontinuing breastfeeding due to difficulty with lactation (*n* = 8). Sixty-eight mother-infant dyads met final study criteria. Data for all participants in each group (*n* = 34 per group) are reported except for the post-intervention period for the one participant who was enrolled into the LS group and withdrew on Day 26 postnatal. The overall attrition rate for this study was 15%, consistent with probiotic studies in healthy, breastfed, term infants [34, 35]. Based on study compliance assessments, of the 21 desired once-daily servings of *B. infantis*, 94% of the BiLS infants consumed 20–25 daily servings and 6% consumed 13–19 daily servings. No infant received more than one serving per day.

### Maternal characteristics

Maternal age at enrollment (Additional file 3: Table S1), weight gain during pregnancy, time when lactogenesis II ensued (< 72 h), and time when membranes ruptured prior to delivery were not significantly different between the LS and BiLS groups (Additional file 4: Table S2). However, women enrolled in the BiLS group had a higher pre-pregnancy BMI than women enrolled in the LS group (*p* < 0.05). There were significantly (*p* < 0.01) more multiparous women (*n* = 20, BiLS; *n* = 8, LS) in the BiLS group and fewer primiparous women (*n* = 14, BiLS; *n* = 26, LS) compared with the LS group (Additional file 4: Table S2). These differences were not a result of the non-random enrollment of the first fifteen participants.

### Infant characteristics

Infant birth weight, birth length, gestational age at birth, and gender were not different between groups (Table 1). Infant weight was not different between groups at birth, or Days 33 and 61 (Additional file 5: Figure S1).

**Table 1:** Infant baseline characteristics

Infant Baseline Characteristics	BiLS (*n* = 34)		LS (*n* = 34)	
	Mean	SD	Mean	SD
Gestational Age (wk)	39.5	1.2	39.9	1.2
Birth Weight (g)	3457.8	369.5	3555.6	624.1
Infant Birth Length (cm)	50.5	2.0	50.6	2.8
Infant Gender, % (*n*)				
Male	62% (21)		44% (15)	
Female	38% (13)		56% (19)	
Medical Complications at Birth Reported, % (*n*)				
Yes	0% (0)		18% (6)	
No	100% (34)		82% (28)	
Oral or IV Antibiotics Use First 72 Hours Postnatal, % (*n*)				
Yes	0% (0)		3% (1)	
No	100% (34)		97% (33)	
Vitamin K Shot Received, % (*n*)				
Yes	97% (33)		88% (30)	
No	3% (1)		12% (4)	
Hepatitis B Vaccine Received, % (*n*)				
Yes	62% (21)		65% (22)	
No	38% (13)		35% (12)	
Colostrum Consumption Postnatal, % (*n*)				
Within 1 h of delivery	68% (23)		50% (17)	
Between 1 and 3 h of delivery	24% (8)		35% (12)	
Between 3 and 6 h of delivery	3% (1)		15% (5	
Between 6 and 12 h of delivery	6% (2)		0% (0)	
Infant Formula Consumption First 72 Hours Postnatal, % (*n*)				
Yes	0% (0)		9% (3)	
No	100% (0)		91% (31)	
Bath First 72 Hours Postnatal, % (*n*)				
Yes	79% (27)		79% (27)	
No	21% (7)		18% (6)	
Unsure	0% (0)		3% (1)	

### Infant diet

According to maternal reports, the mean number of breastfeeds at the breast or with breast milk bottles by their infants was the same for intervention groups at each time period (Additional file 6: Figure S2). The number of days, number of infants who were mixed-fed (consumed some amount of infant formula), or the mean amount of infant formula consumed were not significantly different between the BiLS and LS groups (Additional file 7: Table S3). One mother in the BiLS group and two mothers in the LS group reported feeding her infant non-study probiotics during the post-intervention period (Additional file 7: Table S3). The intake of vitamin D by infants was not different between the intervention groups (data not shown).

Parity did not influence any of the feeding variables except for vitamin D intake during the intervention and post-intervention periods (*p* < 0.01 for both). Primiparous women fed their infants vitamin D 30% of the intervention period and 38% of the post-intervention period compared with multiparous mothers feeding only 7% and 6% of the intervention periods, respectively. Additionally, infant intake of vitamin D significantly *(p* < 0.01) increased over time for both BiLS and LS groups in primiparous (*p* < 0.0005) but not multiparous mothers.

### Infant gastrointestinal health and tolerability

**Fig. 2:**
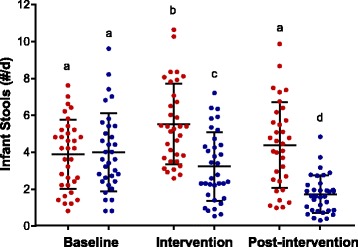
Number of infant stools per day. Mean ± SD of reported number of daily infant stools for the LS (*red dot plot*) and BiLS (*blue dot plot*) groups during the Baseline, Intervention, and Post-intervention periods. *n* = 34 for each group during the Baseline and Intervention periods, and *n* = 33 for the LS, and *n* = 34 for the BiLS groups during the Post-intervention period. Different superscripts represent significant differences within and between interventions. There was a significant time effect (*p* < 0.01), time*trt interaction (*p* < 0.0005), and intervention effect (*p* < 0.0005). Based on multiple comparison post hoc analysis with Bonferroni corrections, compared with baseline the mean number of stools increased (^b^ *p* < 0.0005) during the intervention period for the LS group and decreased (^c^ *p* < 0.05) for the BiLS group. During the Post-intervention period, the mean number of stools returned to Baseline levels for the LS group and decreased from the Intervention period for the BiLS group (^d^ *p* < 0.0005)

**Fig. 3:**
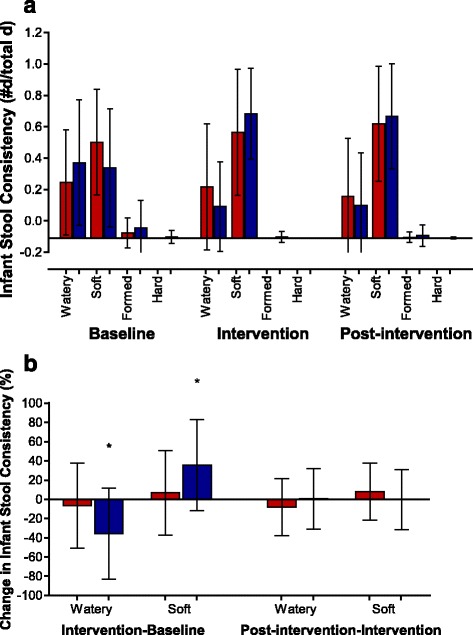
Infant stool consistency. **a** Mean ± SD of the proportion in reported infant stool consistency for the LS (red dot plot) and BiLS (*blue dot plot*) groups during the Baseline, Intervention, and Post-intervention periods. *n* = 34 for each group during the Baseline and Intervention periods, *n* = 33 for the LS group, and *n* = 34 for the BiLS group during the Post-intervention period. **b** Mean ± SD of the change in the percentage of reported infant stool consistency for the LS (red dot plot) and BiLS (*blue dot plot*) groups for difference between Intervention and Baseline (Intervention – Baseline), and Post-intervention and Intervention (Post-intervention – Intervention) periods. *n* = 34 for each group for Intervention – Baseline, and *n* = 33 for the LS and *n* = 34 for the BiLS groups for Post-intervention – Intervention. *p < 0.05

The number of infant bowel movements during the baseline period was the same for the BiLS and LS groups but was significantly (*p* < 0.0005) different during the intervention (BiLS: mean, 3.2/d, range, 0.50–7.2; LS: mean, 5.5/d, range, 2.6–10.6), and post-intervention (BiLS: mean, 1.7/d, range, 0.30–4.8); LS: mean, 4.4/d, range, 0.97–9.9) periods (Fig. 2). The mean number of bowel movements was not only different between groups (*p* < 0.01) but also different across time within each group (*p* < 0.0005). Parity was unrelated to the reported mean number of bowel movements per day across all three time periods. Maternal reports for the proportion of watery and soft stools during the intervention period for infants in the BiLS vs. the LS group (0.20 vs. 0.33) and (0.79 vs. 0.67), respectively, were not statistically significant (Fig. 3a). Yet, the percentage of watery stools decreased from baseline to the intervention period by 36% in infants assigned to the BiLS group (*p* < 0.05) and only by 7% in infants assigned to the LS group. As expected, the percentage of soft stools increased from baseline to the intervention period by 36% in infants assigned to the BiLS group (*p* < 0.05) but only increased by 7% in infants assigned to the LS group (Fig. 3b). There was no difference in the change in consistency from intervention to post-intervention between the groups. Stool consistency was also not influenced by parity.

**Fig. 4:**
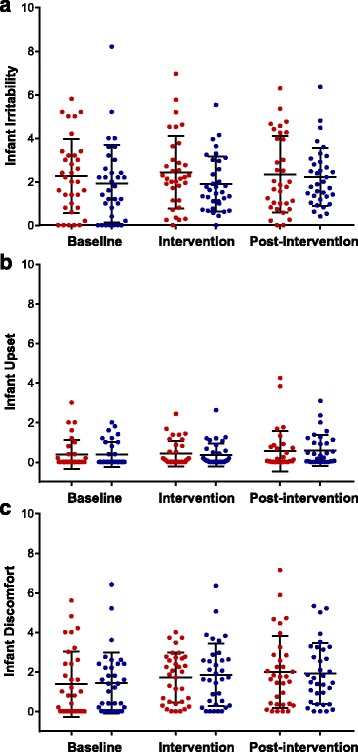
Infant tolerability scores. Mean ± SD of reported tolerability scores (*red dot plot*) and BiLS (blue dot plot) groups during the Baseline, Intervention, and Post-intervention periods. *n* = 34 for each group during the Baseline and Intervention periods, and *n* = 33 for the LS and *n* = 34 for the BiLS groups during the Post-intervention period. **a** Infant irritability, (**b**) infant upset after spit-ups, and (**c**) infant discomfort in passing gas or stool

Infant illness and adverse events were not different between BiLS and LS groups (Table 2). The types of illnesses and reasons for any sick doctor visits reported by mothers are shown in Additional file 8: Table S4. None of the infants in the BiLS group received antibiotics; two infants in the LS group received intravenous antibiotics (at birth and Days 10–18). The number of spit-ups per day as less than five, five to ten, and more than ten were not significantly different at any time period between the two groups (Additional file 9: Figure S3). There were no differences in mean irritability scores (Fig. 4a), mean scores for discomfort after spit-ups (Fig. 4b), or mean scores for discomfort when passing gas or stool (Fig. 4c) between the groups at any time period. Interestingly, maternal parity was significantly associated with rating of irritability. Higher irritability scores were rated by primiparous than multiparous mothers during the baseline period (2.6 and 1.3, respectively, *p* < 0.01) and intervention period (2.5 and 1.7, respectively, *p* < 0.05) but not during the post-intervention period. There were no differences in infant flatulence between groups (Additional file 10: Figure S4).

**Table 2:** Infant tolerability

Tolerability Assessment	BiLS (*n* = 34)						LS (*n* = 33)					
	Baseline		Intervention		Post-Intervention		Baseline		Intervention		Post-Intervention	
	Mean	SD	Mean	SD	Mean	SD	Mean	SD	Mean	SD	Mean	SD
Temperature Above 100.3F, % (# days)^a^	0.005	0.029	0.000	0.000	0.000	0.000	0.005	0.029	0.000	0.000	0.001	0.005
Blood in Stool, % (# days)^a^	0.000	0.000	0.003	0.016	0.002	0.010	0.000	0.000	0.000	0.000	0.005	0.031
Antibiotic Use, % (# days)^a^	0.000	0.000	0.000	0.000	0.000	0.000	0.000	0.000	0.013	0.073	0.000	0.000
Medication for Gas, % (# days)^a^	0.015	0.086	0.038	0.088	0.084	0.194	0.005	0.029	0.059	0.167	0.142	0.254
Jaundice diagnosis, % (*n*)^b^	26.5%	(9)	5.9%	(2)	0%	(0)	26.5%	(9)	8.8%	(3)	2.9%	(1)
Colic, Parental Report, % (*n*)^b^	0%	(0)	0%	(0)	5.9%	(2)	5.9%	(2)	8.8%	(3)	8.8%	(3)
Eczema diagnosis, % (*n*)^b^	0%	(0)	0%	(0)	5.9%	(2)	0%	(0)	0%	(0)	8.8%	(3)
Illnesses, % (# reports)^b^	2.9%	(1)	11.8%	(4)	20.6%	(7)	2.9%	(1)	8.8%	(3)	17.6%	(6)
Sick Doctor Visits, % (# reports)^b^	2.9%	(1)	15%	(5)	15%	(5)	0%	(0)	2.9%	(1)	12%	(4)

^a^Proportions were calculated as: (number of days reported)/total number of days in each study period

^b^Percentages were calculated as: (number of infants for which condition appeared or was diagnosed) /total number of infants in each intervention group during each study period*100

### Fecal *Bifidobacterium*

To correlate safety endpoints with the supplementation of *B. infantis* EVC001 and colonization of the genus *Bifidobacterium* in the infant gut, we compared the mean differences for total fecal *Bifidobacterium* from baseline to the end of the supplementation period for the LS and BiLS groups. The mean Log_10_ change in total fecal *Bifidobacterium* from Day 6 to Day 28 was significantly (*p* = 0.0002) higher for infants in the BiLS group (6.6 ± 2.8 SD) compared with infants in the LS group (3.5 ± 3.5 SD). The median Log_10_ change from Day 6 to Day 28 for total fecal *Bifidobacterium* was median 0.0 for infants in the LS group and 7.5 for infants in the BiLS group (*p* = 0.0002).

## Discussion

The hygiene hypothesis suggests that changes in colonizing microbes related to a developed or Western lifestyle have long-term impacts on the risks of developing allergic, inflammatory, and autoimmune diseases [21–28]. In an effort to shift the intestinal microbiome toward beneficial populations in early infancy, the Infant Microbiota Probiotic Intake (IMPRINT) Study was designed to determine the safety and tolerability in breastfed term infants supplemented with *B. infantis*. We found that *B. infantis* EVC001 was well-tolerated and safely consumed by healthy term infants for 21 consecutive days. All adverse events reported by mothers enrolled in the study were typical of infants this age and the incidences were not increased by the *B. infantis* feedings.

To assess infant GI function as a metric of tolerability, mothers recorded the number of bowel movements and the consistency of the first bowel movement produced by their infants each day during the two-month study period. Based on daily reports, the mean number of bowel movements passed by infants in both groups are consistent with other reports on the frequency of bowel movements in breastfed infants. The number of bowel movements in breastfed infants has been found to be highly variable and to decrease with postnatal age, with a mean of about 4 per day and a median of about 4 per day and a range of 0.3 to 8.5 per day during the first month of age [36–40]; and a mean of 1.8 per day and median of 1.8 per day at 5 months of age [36–42]. Thus, the frequency in bowel movements of infants in the LS and BiLS groups was within normal a range.

The infants in the BiLS group passed fewer daily stools than infants in the LS group. Additionally, infants in the BiLS group were reported to pass “soft” stools more often and “watery” stools less often compared with infants in the LS group. These data may reflect maturation of the gut in response to *B. infantis* supplementation. Compared with other reports, the LS group produced bowel movements more similar to one-month-old infants, and the BiLS group produced bowel movements more similar to three-month-old infants [36, 38]. In infants, a reduction in stool frequency and increase in stool firmness has been found to be associated with postnatal age and with the maturation of the gut [32, 36, 38]. In addition to infant age and diet [36, 39], stool consistency was recently reported to be associated with the intestinal bacterial species richness, enterotypes (or microbial classification), and community composition in adults [43]. We found a 1000-fold higher change in total fecal *Bifidobacterium* from baseline to the end of the supplementation period in infants supplemented with *B. infantis* EVC001 compared with unsupplemented infants. The changes in total fecal *Bifidobacterium* are biologically relevant and can logically be attributed to the supplementation of *B. infantis* EVC001. We hypothesize that supplementation of infants with *B. infantis* facilitates maturation of the gut mucosa. This hypothesis is supported by the findings that intestinal *Bifidobacterium* and *B. infantis* increase the mRNA expression of intestinal epithelial tight junction proteins [15], enhance intestinal barrier function through the production of acetate [44], and promote the maturation of dendritic cells in intestinal Peyer’s Patches [45].

To investigate overall GI tolerability, mothers were asked to rate their infants’ general irritability, upset feelings after spitting-up, and discomfort when passing gas or stool on a daily basis throughout the two-month study period. Generally, reports of infants’ general irritability, upset feelings, and discomfort scores were low and not different between the groups. Interestingly, maternal parity was significantly associated with rating irritability during the baseline and intervention but not the post-intervention period. Mean infant irritability scores were higher by primiparous than multiparous mothers, reflecting an over-assessment of infant behavior in first-time mothers [46]. The number of days blood in stool was present, number of daily spit-ups, and daily flatulence frequency were not different between the groups. Adverse events occurred in both groups based on evaluation of the presence of colic, number of sick doctor visits, illnesses, eczema diagnoses by a primary care providers, and use of antibiotics or gas-relieving medications. The types of events, however, were normal for infants of this age, were not serious in nature, and the incidence of adverse events was not greater in the BiLS group than in the LS group. The adverse events were not deemed related to the study procedures or feeding of *B. infantis*.

One limitation of our study was that a placebo was not supplied for the control arm and subjects were not blinded to their treatment assignment. Although these measures are critical for larger efficacy studies, they are not imperative for small Phase I trials. At the time the study was initiated, a placebo was not feasible, however we have recently identified a safe placebo that would not elicit a prebiotic effect in infants and feasible for future trials. Another limitation is a potential source of bias with the involvement of the clinical coordinator in allocating group assignments at enrollment. This was due to limited resources in using a neutral third party for randomization. Bias was checked weekly by the principal investigator to ensure the clinical coordinator had allocated group assignments in chronological order and according to the randomization scheme that was created prior to study initiation. Another limitation was that mothers were instructed to assess stool consistency by comparing their infants’ bowel movements with images of four typical stool consistencies. Assessing stool consistency by appearance using a 4-point system is subjective, and more objective metrics of stool quality are needed in the clinical setting. Furthermore, in contrast with studies in adolescents [47], the infant stool scale used in this study has not been correlated with colonic transit time [32], making interpretation with GI function challenging. Another limitation was that mothers were not instructed to report the amount of infant stool produced, which has been found to be a reliable metric of GI maturation [32]. We did not include bowel movement size because of difficulty in assessing the amount of stool as a percentage of total surface on the diaper due to the excellent absorbency of today’s disposable diapers. Lastly, balancing the number of primiparous and multiparous women would have better controlled confounding effects of assessing infant symptoms.

## Conclusions

In this study of normal, healthy, term infants, supplementation of *B. infantis* EVC001 for 21 consecutive days in maternal breast milk was well-tolerated and increased total infant fecal *Bifidobacterium*. There was no difference in the number or type of reported adverse events between supplemented and non-supplemented infants.

## Additional files

Additional file 1: Infant Stool Scale. Stool consistency rating scale from the validated Amsterdam Scale that was used in this study. (PDF 81 kb)

Additional file 2: Weekly Health Questionnaire. The weekly health questionnaire used in this study that mothers filled out each week or every two weeks. (PDF 495 kb)

Additional file 3: Table S1. Maternal baseline demographics. (DOCX 20 kb)

Additional file 4: Table S2. Baseline maternal pregnancy and related characteristics. (DOCX 21 kb)

Additional file 5: Figure S1. Mean ± SD of reported infant birthweight and infant weight measured on Days 33 and 61 postnatal for the LS (red dot plot) and BiLS (blue dot plot). *n* = 34 for each group for birthweight, *n* = 33 for the LS, and *n* = 34 for the BiLS groups on Days 33 and 61 postnatal. (DOCX 91 kb)

Additional file 6: Figure S2. Mean ± SD of reported number of infant breast milk intake at the breast and by bottle for the LS (red dot plot) and BiLS (blue dot plot) groups during the Baseline, Intervention, and Post-intervention periods. *n* = 34 for each group during the Baseline and Intervention periods, *n* = 33 for the LS, and *n* = 34 for the BiLS groups during the Post-intervention period. (DOCX 91 kb)

Additional file 7: Table S3. Infant diet throughout the study period. (DOCX 19 kb)

Additional file 8: Table S4. Maternal reports of infant illnesses and reasons for infant sick-doctor visits throughout the study period. (DOCX 21 kb)

Additional file 9: Figure S3. Mean ± SD of the change in the proportion of infant spit-ups for the LS (red dot plot) and BiLS (blue dot plot) groups during the Baseline, Intervention, and Post-intervention periods. *n* = 34 for each group during the Baseline and Intervention periods, and *n* = 33 for the LS and *n* = 34 for the BiLS groups during the Post-intervention period. (DOCX 51 kb)

Additional file 10: Figure S4. Mean ± SD of the proportion of infant flatulence reported by mothers (red dot plot) and BiLS (blue dot plot) groups during the Baseline, Intervention, and Post-intervention periods. *n* = 17 for the LS and *n* = 12 for the BiLS groups for all time periods. (DOCX 54 kb)

## Abbreviations

ANOVA: Analysis of variance
*B. infantis*: *Bifidobacterium longum* subspecies *infantis*
BiLS: Lactation support plus *B. infantis* supplementation
GI: Gastrointestinal
HMO: Human milk oligosaccharides
IBCLC: internationally board certified lactation consultant
IMPRINT: Infant microbiota probiotic intake study
IRB: Institutional Review Board
LS: Lactation support alone
PCR: Polymerase chain reaction
UC: University of California
