# Erratum to: Safety and tolerability of Bifidobacterium longum subspecies infantis EVC001 supplementation in healthy term breastfed infants: a phase I clinical trial

**DOI:** 10.1186/s12887-017-0932-7

**Published:** 2017-08-15

**Authors:** Jennifer T. Smilowitz, Jackelyn Moya, Melissa A. Breck, Chelsea Cook, Annette Fineberg, Kathleen Angkustsiri, Mark A. Underwood

**Affiliations:** 1 Department of Food Science and Technology, University of California, Davis, CA USA; 2 Foods for Health Institute, University of California, One Shields Ave, Davis, CA 95616 USA; 3 Sutter Health, Sutter Davis Hospital, Davis, CA USA; 4 UC Davis MIND Institute, University of California, Davis, CA USA; 5 Department of Pediatrics, UC Davis Children’s Hospital, Sacramento, CA USA

## Erratum

Following the publication of this article [1], it was brought to our attention that the lower SD bars in Fig. 3a were unfortunately omitted. Furthermore, the bar graphs were erroneously referred to as “dot plots” in the figure legend. The correct Fig. 3a is presented below:

**Fig. 3:**
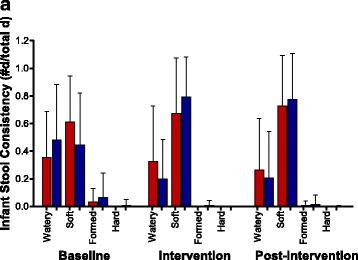
Infant stool consistency. **a** Mean + SD of the proportion in reported infant stool consistency for the LS (*red bar*) and BiLS (*blue bar*) groups during the Baseline, Intervention, and Post-intervention periods. *n* = 34 for each group during the Baseline and Intervention periods, *n* = 33 for the LS group, and *n* = 34 for the BiLS group during the Post-intervention period

The above have been updated in the original version of the article.
