# Supplementary material for: Safety and tolerability of Bifidobacterium longum subspecies infantis EVC001 supplementation in healthy term breastfed infants: a phase I clinical trial
Source: BMC Pediatr. 2017 May 30;17:133. doi: 10.1186/s12887-017-0886-9 (PMC5450358; doi:10.1186/s12887-017-0886-9)
Supplement: Supplementary file 1 — Infant Stool Scale. Stool consistency rating scale from the validated Amsterdam Scale that was used in this study. (PDF 81 kb) [file 12887_2017_886_MOESM1_ESM.pdf]

UC DAVIS
FOODS FOR HEALTH INSTITUTE

IMPRINT Study

Infant Stool Form Scale

Consistency

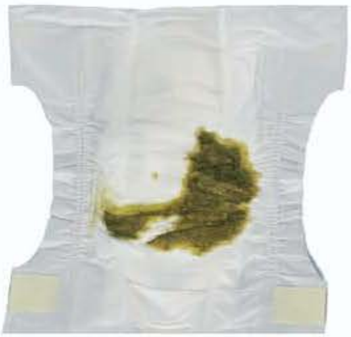

A: watery

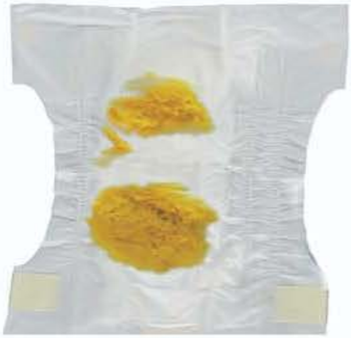

B: soft

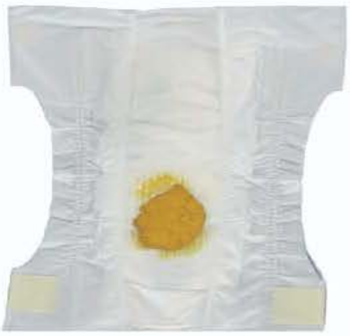

C: formed

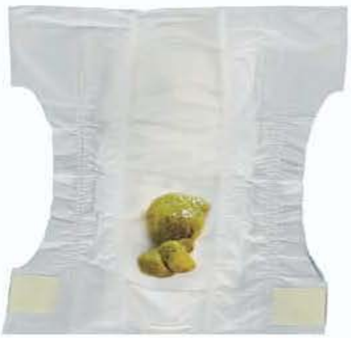

D: hard

Please use this Infant Stool Scale to rate your baby's FIRST stool of the day on your Daily Infant Gut Symptomology Log.

Please use the designated “A”, “B”, “C”, or “D” scores found below each image to rate the consistency of your baby's stool.

Please use your best judgment when rating the stool consistency.

If your baby's stool falls between two categories, select the rating that best describes the stool.

For example, if your baby's stool is partly formed (C) but mostly still soft (B), please write “B” on your Daily Infant Gut Symptomology Log.
