# Supplementary material for: Safety and tolerability of Bifidobacterium longum subspecies infantis EVC001 supplementation in healthy term breastfed infants: a phase I clinical trial
Source: BMC Pediatr. 2017 May 30;17:133. doi: 10.1186/s12887-017-0886-9 (PMC5450358; doi:10.1186/s12887-017-0886-9)
Supplement: Supplementary file 2 — Weekly Health Questionnaire. The weekly health questionnaire used in this study that mothers filled out each week or every two weeks. (PDF 495 kb) [file 12887_2017_886_MOESM2_ESM.pdf]

Subject ID: 7XXX

Weekly Infant and Maternal Health Questionnaire

EXAMPLE: END OF WEEK 1: Day 7

Please answer the following questions regarding you and your infant's health at the end of each week. Please circle your answers, and answer any follow-up questions as appropriate. We will not share your answers with anyone. If you prefer not to answer a question, you may circle "refuse". Please refer to the example questionnaire in paper form included in your study notebook as a guide with example answers written in red.

DATE: *7/30/2014*

Study Personnel Section:
Actual Day of Life: _______ **Initials:** ________

Questions about your baby

1. In the **past week**, has your baby shown signs of **COLIC?** YES NO UNSURE REFUSE
[A colicky baby is defined as one that cries for more than 3 hours per day, for at least 3 days per week]

a. If "**YES**" to question 1, when did the colic appear? ______*7/23/2014*__ OR **UNSURE** **REFUSE**
month/day/year

b. If **"YES"** to question 1, did your baby receive treatment for colic? **YES** **NO** **REFUSE**

c. If **"YES"** to question 1b, how was your baby treated for colic? ***Fed baby gripe water and simethicone***

d. If "**YES**" to question 1, does your baby still have colic? **YES** **NO** **UNSURE** **REFUSE**

e. If "**NO**" to question 1d, when did the colic go away? ______*7/29/2014*_____ OR **UNSURE** **REFUSE**
month/day/year

2. In the **past week**, has your baby been diagnosed with **ECZEMA** by a healthcare provider?
[Eczema shows up as patches of red skin. The skin is almost always itchy, dry, and rough and most often occurs on a baby's cheeks and at the joints of their arms and legs].
YES **NO** **REFUSE**

a. If "**YES**" to question 2, when was the eczema diagnosed? _______*7/25/2014*__ OR **UNSURE** **REFUSE**
month/day/year

b. Where was the eczema found on your baby's body? ***Left and right elbows, behind left knee***

c. Did your baby receive treatment for eczema? **YES** **NO** **REFUSE**

d. If "**YES**" to question 2c, how was your baby treated for eczema? ***Eucerin Baby Eczema Relief Moisturizer***

e. As of today, what is the current status of your baby's eczema (please circle one)?
Severe **Moderate** **Mild** **Gone**

f. If the eczema has gone, when did it go away? _______*7/25/2014*_____ OR **UNSURE** **REFUSE**
month/day/year

3. In the **past week**, has your baby been diagnosed with **JAUNDICE** by a healthcare provider? **YES** **NO** **REFUSE**

a. If "**YES**" to question 3, when was the jaundice diagnosed? _______*7/25/2014*__ OR **UNSURE** **REFUSE**
month/day/year

b. If **"YES"** to question 3, did your baby receive treatment for jaundice? **YES** **NO** **REFUSE**

Weekly Infant and Maternal Health Questionnaire

c. If "**YES**" to question 3b, how was your baby treated for jaundice? ***Phototherapy***

d. If "**YES"** to question 3, does your baby still have jaundice? **YES NO UNSURE REFUSE**

e. If "**NO"** to question 3d, when did the jaundice go away? ______***7/23/2014***____ OR **UNSURE REFUSE**
month/day/year

4. In the **past week**, did your baby have any **SICK-child DOCTOR VISITS**? **YES NO REFUSE**
[A sick-child doctor visit is an appointment the parent makes when the baby is not feeling well].

a. If "**YES**", when did your baby have the sick-doctor visit? ______***7/24/2014***_____
month/day/year

b. If "**YES**", what was the reason for the sick-child doctor visit (please explain)? ***To monitor his jaundice with a blood test for bilirubin levels and ask the doctor about the eczema on his back, elbows and knee.***

5. In the **past week**, has your baby shown signs of any **ILLNESSES**? **YES NO REFUSE**

a. If "**YES**", please describe the illness (*examples: cold, flu, ear infection, etc*.). ***Cold with fever***

b. Did your baby consume any medications to treat the illness(s)? **YES NO REFUSE**

c. If "**YES**" to question 5b, please list the medications your baby consumed in the **past week**: ***Baby Motrin***

6. In the **past week**, did your baby have a **WELL-child DOCTOR VISIT**? **YES NO REFUSE**
[A well-child doctor visit is a routine appointment that gives the doctor a chance to look at your baby's overall health].

a. If "**YES**" to question 6, when did your baby have the well-child doctor visit? ______***7/24/2014***_____
month/day/year

b. Were any measurements taken during this visit? **YES NO REFUSE**

c. If "**YES**" to questions 6b, please list the measurements taken (or write "none" for any measurements that were not taken).

Infant length: ___***21.25***___
inches

Infant weight: ___***9 pounds, 4 ounces***_________
Pounds & ounces

Infant head circumference: ______***14.1***______
inches

d. Please describe any medical problems reviewed by your baby's doctor during this visit (if no medical problems were reviewed, please write "none"): ______***None***_________

7. In the **past week**, did your baby have any baths at home between **hospital discharge and Day 7 of life**?
YES NO REFUSE

a. If "**YES**", how many baths did your baby have at home between **hospital discharge and Day 7 of life**?
# of baths:__***3***____

b. If your baby had at least one bath in question 7, for how many baths was soap used?
[This includes any type of soap, including body gel and shampoo]: **# of soapy baths:**__***1***_

c. Please list the brand(s) of soap used. ***California Baby Calming Shampoo and Body Wash***___________

Questions about you

Weekly Infant and Maternal Health Questionnaire

8. In the **past week**, have **you** experienced any inflammation in your breasts? **YES** **NO** **UNSURE** **REFUSE**
[Inflammation of your breast includes mastitis, blocked duct, bacterial infection, dermatitis, milk bleb, vasospasm, ductal infection or yeast]

If **"YES",** please fill out the ***Inflammation Survey*** found in your Study Notebook.

9. Have **you** consumed any of the following in the **past week** (please circle all that apply)?

Antibiotics (circled) **Probiotic supplements** (circled) **Yogurt containing bifidobacteria** **None**

10. If you circled **ANTIBIOTICS** and/or **PROBIOTIC SUPPLEMENTS**, please describe the name of the product(s), start and end dates, and the reason for taking them.

Product name	Start Date	End Date	Reason for taking
Ex: dicloxacillin	*July 24, 2014*	*Still taking*	*To treat mastitis*
Ex: VSL #3	*January 1, 2014*	*Still taking*	*To improve gut health*

11. In the **past week**, did you experience any **ILLNESSES**? **YES** (circled) **NO** **REFUSE**

a. If "**YES**", please describe the illnesses (*examples: cold, flu, etc*.): ***mastitis and a cold***

12. How many servings of scaly fish did you eat this **past week** (one 3 ounce serving of fish is the size of a deck of cards)? **# of servings:**____*2*___

13. How many servings of shellfish did you eat this **past week** (one 3 ounce serving of fish is the size of a deck of cards)? **# of servings:** ____*0*____

14. Have you taken any fish oil in the **past week**? **YES** (circled) **NO** **REFUSE**

a. If yes, please list the brand and product name of fish oil, dose consumed per day, and number of days this week you consumed it.

Fish Oil Brand	Product Name	Amount/d	How many days per week do you take the daily dose?

Weekly Infant and Maternal Health Questionnaire

Ex: Nordic Naturals	*DHA Extra*	*2 capsules*	*7*

15. Have you taken any other vitamins or supplements in the **past week**? **YES** (circled) **NO** **REFUSE**

a. If yes, please mark all that apply:

- ☐ Beta Carotene
- ☐ *X* B complexes (stress tabs)
- ☐ *X* Calcium
- ☐ Co-enzyme Q10
- ☐ Flaxseeds, flaxseed meal, flaxseed oil or chia seeds
- ☐ Folic acid
- ☐ Ginseng
- ☐ Ginko biloba
- ☐ *X* Iron
- ☐ Multivitamin **with iron**
- ☐ Multivitamin **without iron**
- ☐ Niacin
- ☐ Prenatal vitamin **with iron**
- ☐ *X* Prenatal vitamin **without iron**
- ☐ Selenium
- ☐ St. John's Wort
- ☐ Vitamin A/Retinol
- ☐ Vitamin B1
- ☐ Vitamin B6
- ☐ Vitamin B12
- ☐ Vitamin C
- ☐ *X* Vitamin D
- ☐ Vitamin E
- ☐ Vitamin K
- ☐ *X* Zinc
- ☐ Other (please list): ______________________________

Weekly Infant and Maternal Health Questionnaire
