# Supplementary material for: Safety and tolerability of Bifidobacterium longum subspecies infantis EVC001 supplementation in healthy term breastfed infants: a phase I clinical trial
Source: BMC Pediatr. 2017 May 30;17:133. doi: 10.1186/s12887-017-0886-9 (PMC5450358; doi:10.1186/s12887-017-0886-9)
Supplement: Supplementary file 3 — Maternal baseline demographics. (DOCX 20 kb) [file 12887_2017_886_MOESM3_ESM.docx]

**Table S1** Maternal baseline demographics

| Maternal Baseline Characteristics | BiLS (*n* = 34) | | LS (*n* = 34) | |
| --- | --- | --- | --- | --- |
|  | Mean | SD | Mean | SD |
| Age at Enrollment (yr) | 33.3 | 4.5 | 31.4 | 3.5 |
| Ethnicity, % (n) |  |  |  |  |
| Not Hispanic | 82% (28) | | 94% (32) | |
| Hispanic | 18% (6) | | 6% (2) | |
| Race, % (n) |  |  |  |  |
| Asian | 0% (0) | | 9% (3) | |
| Black | 0% (0) | | 3% (1) | |
| White | 76% (26) | | 79% (27) | |
| Other | 6% (2) | | 0% (0) | |
| 2 or More Races | 18% (6) | | 9% (3) | |
| Education, % (n) |  |  |  |  |
| Some College, No Degree; or AA Degree | 18% (6) | | 15% (5) | |
| Bachelor's Degree (BA or BS) | 26% (9) | | 32% (11) | |
| Secondary Degree or Professional School (MS, PhD, MD, etc) | 56% (19) | | 53% (18) | |
| Blood Type, % (n) |  |  |  |  |
| A+ | 32% (11) | | 35% (12) | |
| A- | 15% (5) | | 3% (1) | |
| B+ | 9% (3) | | 12% (4) | |
| B- | 0% (0) | | 0% (0) | |
| AB+ | 6% (2) | | 6% (2) | |
| AB- | 3% (1) | | 0% (0) | |
| O+ | 24% (8) | | 35% (12) | |
| O- | 12% (4) | | 9% (3) | |
| Any Allergy Diagnosis in Past 10 Years, % (n) |  |  |  |  |
| Yes | 29% (10) | | 29% (10) | |
| No | 71% (24) | | 71% (24) | |
| Asthma Diagnosis in Past 10 Years, % (n) |  |  |  |  |
| Yes | 12% (4) | | 15% (5) | |
| No | 88% (30) | | 85% (29) | |
| Hay Fever Diagnosis in Past 10 Years, % (n) |  |  |  |  |
| Yes | 9% (3) | | 3% (1) | |
| No | 91% (31) | | 97% (33) | |
| Use of Oral or IV Antibiotics in Past 10 Years (# of courses), % (n) |  |  |  |  |
| 0 Courses | 12% (4) | | 9% (3) | |
| 1-3 Courses | 62% (21) | | 47% (16) | |
| 4-6 Courses | 12% (4) | | 29% (10) | |
| 7-10 Courses | 6% (2) | | 12% (4) | |
| 10-15 Courses | 9% (3) | | 3% (1) | |
| Sentiments about using Products Containing Antimicrobials % (n) |  |  |  |  |
| Tries to Avoid Antimicrobials | 29% (10) | | 35% (12) | |
| Prefers to Use Them Some of the Time | 26% (9) | | 32% (11) | |
| Prefers to Use Them at All Times | 0% (0) | | 0% (0) | |
| No Preference | 32% (11) | | 21% (7) | |
| Other | 12% (4) | | 12% (4) | |
