# Supplementary material for: Safety and tolerability of Bifidobacterium longum subspecies infantis EVC001 supplementation in healthy term breastfed infants: a phase I clinical trial
Source: BMC Pediatr. 2017 May 30;17:133. doi: 10.1186/s12887-017-0886-9 (PMC5450358; doi:10.1186/s12887-017-0886-9)
Supplement: Supplementary file 4 — Baseline maternal pregnancy and related characteristics. (DOCX 21 kb) [file 12887_2017_886_MOESM4_ESM.docx]

**Table S2** Baseline maternal pregnancy and related characteristics

| Maternal Pregnancy & Labor Baseline Characteristics | BiLS (*n* = 34) | | LS (*n* = 34) | |
| --- | --- | --- | --- | --- |
|  | Mean | SD | Mean | SD |
| Pre-Pregnancy BMI^a^ | 25.6 | 3.6 | 23.8 | 3.2 |
| Pregnancy Weight Gain (kg) | 15.0 | 5.1 | 15.2 | 5.0 |
| Hours in Labor (hr) | 12.9 | 12.8 | 17.8 | 22.3 |
| Ruptured Membranes Prior to Birth (hr) | 10.1 | 17.2 | 10.8 | 16.2 |
| Onset of Lactogenesis II (hr postnatal) | 61:09:21 | 17:57:27 | 66:50:22 | 17:25:02 |
| Number of Pregnancies^b^ | 2.7 | 1.6 | 1.8 | 1.3 |
| Number of Live Births^b^ | 2.0 | 1.0 | 1.4 | 0.8 |
| Parity, % (n)^b^ |  |  |  |  |
| Primiparous | 41% (14) | | 76% (26) | |
| Multiparous | 59% (20) | | 24% (8) | |
| Delivery Location |  |  |  |  |
| Hospital or Birthing Center | 94% (32) | | 97% (33) | |
| Home Birth | 6% (2) | | 3% (1) | |
| Mode of Delivery, % (n) |  |  |  |  |
| Vaginal | 68% (23) | | 56% (19) | |
| Vaginal Water Birth | 0% (0) | | 15% (5) | |
| C-section, Elective | 12% (4) | | 15% (5) | |
| C-section, Emergent | 21% (7) | | 15% (5) | |
| Medication Use Labor, % (n) |  |  |  |  |
| Analgesics, narcotics | 3% (1) | | 3% (1) | |
| Epidural anesthesia | 32% (11) | | 29% (10) | |
| Spinal block | 6% (2) | | 12% (4) | |
| None | 41% (14) | | 41% (14) | |
| Other | 18% (6) | | 15% (5) | |
| Antibiotic Use Labor, % (n) |  |  |  |  |
| Yes | 44% (15) | | 29% (10) | |
| No | 56% (19) | | 71% (24) | |
| Labor Complications Reported, % (n) |  |  |  |  |
| Yes | 26% (9) | | 26% (9) | |
| No | 74% (25) | | 74% (25) | |
| Gestational Diabetes Mellitus Diagnosis, % (n) |  |  |  |  |
| Yes | 9% (3) | | 9% (3) | |
| No | 91% (31) | | 91% (31) | |
| GBS Positive Diagnosis, % (n) |  |  |  |  |
| Yes | 26% (9) | | 18% (6) | |
| No | 74% (25) | | 82% (28) | |
| Other Diagnosed Infections (Non-GBS), % (n) |  |  |  |  |
| Yes | 24% (8) | | 15% (5) | |
| No | 76% (26) | | 85% (29) | |
| Number of Other Diagnosed Infections, % (n) |  |  |  |  |
| No Infections | 76% (26) | | 85% (29) | |
| 1 Infection | 15% (5) | | 6% (2) | |
| 2 Infections | 3% (1) | | 3% (1) | |
| 3 Infections | 6% (2) | | 6% (2) | |
| Antibiotic Use 1-Year Before and During Pregnancy, % (n) |  |  |  |  |
| Yes | 24% (8) | | 18% (6) | |
| No | 76% (26) | | 82% (28) | |
| Number of Antibiotic Courses 1-Year Before and During Pregnancy, % (n) | |  |  |  |
| No Antibiotic Courses | 76% (26) | | 82% (28) | |
| 1 Course | 12% (4) | | 3% (1) | |
| 2 Courses | 6% (2) | | 9% (3) | |
| 3 Courses | 3% (1) | | 6% (2) | |
| 4 Courses | 3% (1) | | 0% (0) | |
| Probiotic Use 3-Months Before and During Pregnancy, % (n) |  |  |  |  |
| Yes | 26% (9) | | 29% (10) | |
| No | 74% (25) | | 71% (24) | |

^a^ Significantly different between intervention groups, *p* < 0.05.

^b^ Significantly different between intervention groups, *p* < 0.01.
