# Supplementary material for: Safety and tolerability of Bifidobacterium longum subspecies infantis EVC001 supplementation in healthy term breastfed infants: a phase I clinical trial
Source: BMC Pediatr. 2017 May 30;17:133. doi: 10.1186/s12887-017-0886-9 (PMC5450358; doi:10.1186/s12887-017-0886-9)
Supplement: Supplementary file 5 — Mean ± SD of reported infant birthweight and infant weight measured on Days 33 and 61 postnatal for the LS (red dot plot) and BiLS (blue dot plot). n = 34 for each group for birthweight, n = 33 for the LS, and n = 34 for the BiLS groups on Days 33 and 61 postnatal. (DOCX 91 kb) [file 12887_2017_886_MOESM5_ESM.docx]

**Figure S1** **Infant weight.** Mean ± SD of reported infant birthweight and infant weight measured on Days 33 and 61 postnatal for the LS (red dot plot) and BiLS (blue dot plot). *n* = 34 for each group for birthweight, *n* = 33 for the LS, and *n* = 34 for the BiLS groups on Days 33 and 61 postnatal.
