# Supplementary material for: Safety and tolerability of Bifidobacterium longum subspecies infantis EVC001 supplementation in healthy term breastfed infants: a phase I clinical trial
Source: BMC Pediatr. 2017 May 30;17:133. doi: 10.1186/s12887-017-0886-9 (PMC5450358; doi:10.1186/s12887-017-0886-9)
Supplement: Supplementary file 6 — Mean ± SD of reported number of infant breast milk intake at the breast and by bottle for the LS (red dot plot) and BiLS (blue dot plot) groups during the Baseline, Intervention, and Post-intervention periods. n = 34 for each group during the Baseline and Intervention periods, n = 33 for the LS, and n = 34 for the BiLS groups during the Post-intervention period. (DOCX 91 kb) [file 12887_2017_886_MOESM6_ESM.docx]

**Figure S2 Infant breast milk intake**. Mean ± SD of reported number of infant breast milk intake at the breast and by bottle for the LS (red dot plot) and BiLS (blue dot plot) groups during the Baseline, Intervention, and Post-intervention periods. *n* = 34 for each group during the Baseline and Intervention periods, *n* = 33 for the LS, and *n* = 34 for the BiLS groups during the Post-intervention period.
