# Supplementary material for: Safety and tolerability of Bifidobacterium longum subspecies infantis EVC001 supplementation in healthy term breastfed infants: a phase I clinical trial
Source: BMC Pediatr. 2017 May 30;17:133. doi: 10.1186/s12887-017-0886-9 (PMC5450358; doi:10.1186/s12887-017-0886-9)
Supplement: Supplementary file 7 — Infant diet throughout the study period. (DOCX 19 kb) [file 12887_2017_886_MOESM7_ESM.docx]

**Table S3** Infant diet throughout the study period

|  | BiLS (*n* = 34) | | | | | | LS (*n* = 33) | | | | | |
| --- | --- | --- | --- | --- | --- | --- | --- | --- | --- | --- | --- | --- |
|  | Baseline | | Intervention | | Post-Intervention | | Baseline | | Intervention | | Post-Intervention | |
|  | Mean | SD | Mean | SD | Mean | SD | Mean | SD | Mean | SD | Mean | SD |
| Infant Formula Use, (mL/d) | 4.2 | 20.3 | 3.0 | 17.7 | 15.4 | 60.9 | 2.8 | 8.5 | 10.1 | 58.9 | 4.5 | 20.6 |
| Infant Formula Use, %, (# days)^1^ | 0.041 | 0.146 | 0.015 | 0.090 | 0.062 | 0.212 | 0.065 | 0.182 | 0.029 | 0.155 | 0.026 | 0.087 |
| Infant Formula Use, %, (# infants)^2^ | 12% | (4) | 3% | (1) | 9% | (3) | 12% | (4) | 9% | (3) | 12% | (4) |
| Non-study Probiotic Use, % (# days)^1^ | 0.005 | 0.029 | 0.000 | 0.000 | 0.002 | 0.010 | 0.005 | 0.029 | 0.000 | 0.000 | 0.028 | 0.135 |

^1^Proportions were calculated as: (number of days reported)/total number of days in each study period.

^2^Percentages were calculated as: (number of infants who consumed any amount of infant formula)/total number of infants in each intervention group during each study period*100.
