# Supplementary material for: Safety and tolerability of Bifidobacterium longum subspecies infantis EVC001 supplementation in healthy term breastfed infants: a phase I clinical trial
Source: BMC Pediatr. 2017 May 30;17:133. doi: 10.1186/s12887-017-0886-9 (PMC5450358; doi:10.1186/s12887-017-0886-9)
Supplement: Supplementary file 8 — Maternal reports of infant illnesses and reasons for infant sick-doctor visits throughout the study period. (DOCX 21 kb) [file 12887_2017_886_MOESM8_ESM.docx]

**Table S4** Maternal reports of infant illnesses and reasons for infant sick-doctor visits throughout the study period^1^

| BiLS (*n* = 34) | | | | | | |
| --- | --- | --- | --- | --- | --- | --- |
|  | Baseline | | Intervention | | Post-intervention | |
| Subject ID | Illnesses | Reasons for sick Dr. visits | Illnesses | Reasons for sick Dr. visits | Illnesses | Reasons for sick Dr. visits |
| 1 | Fever | NR | NR | NR | NR | NR |
| 2 | NR | (1) Jaundice, (2) vomiting, (3) weight checks^2^ | Cold-nasal congestion | NR | Mild runny nose | Heavy toy dropped on head |
| 3 | NR | NR | NR | Check weight, jaundice, hemoglobin | Cold, congestion | NR |
| 4 | NR | NR | NR | Clogged tear duct | NR | NR |
| 5 | NR | NR | Cold | NR | Cold | NR |
| 6 | NR | NR | NR | Lack of bowel movement for 48+ hours | NR | NR |
| 7 | NR | NR | Failure to thrive | NR | NR | Follow-up for low weight |
| 8 | NR | NR | NR | Enlarged kidneys- had an ultrasound | NR | NR |
| 9 | NR | NR | Conjunctivitis | Conjunctivitis | NR | NR |
| 10 | NR | NR | NR | NR | NR | Reflux, fussiness |
| 11 | NR | NR | NR | NR | Thrush | Excessive spitting up, thrush |
| 12 | NR | NR | NR | NR | Cold | Runny nose, congestion |
| 13 | NR | NR | NR | NR | Cold | NR |
| 14 | NR | NR | NR | NR | Cold | NR |
|  | LS (*n* = 34) | | | | | |
|  | Baseline | | Intervention | | Post-intervention | |
| Subject # | Illnesses | Reasons for sick Dr. visits | Illnesses | Reasons for sick Dr. visits | Illnesses | Reasons for sick Dr. visits |
| 15 | Stuffy nose at nighttime | NR | NR | NR | NR | NR |
| 16 | NR | NR | Eye infection | NR | NR | NR |
| 17 | NR | NR | NR | Tongue clipping for ankyloglossia | NR | Heat rash |
| 18 | NR | NR | NR | NR | Cold, gastroenteritis, diarrhea, painful diaper rash | NR |
| 19 | NR | NR | NR | NR | Cold | NR |
| 20 | NR | NR | NR | NR | Cold | NR |
| 21 | NR | NR | Runny nose | NR | NR | NR |
| 22 | NR | NR | Thrush | NR | Cold | NR |
| 23 | NR | NR | NR | NR | Cold | NR |
| 24 | NR | NR | NR | NR | Cold | NR |
| 25 | NR | NR | NR | NR | NR | Bloody stool |
| 26 | NR | NR | NR | NR | NR | Diaper rash |
| 27 | NR | NR | NR | NR | NR | Colic/fussy |

^1^Mothers reported illnesses their infants experienced and the reasons for infant sick doctor visits. Not reported, NR.

^2^One infant visited a primary care provider on three different occasions to address three separate issues.
