# Supplementary material for: Safety and tolerability of Bifidobacterium longum subspecies infantis EVC001 supplementation in healthy term breastfed infants: a phase I clinical trial
Source: BMC Pediatr. 2017 May 30;17:133. doi: 10.1186/s12887-017-0886-9 (PMC5450358; doi:10.1186/s12887-017-0886-9)
Supplement: Supplementary file 9 — Mean ± SD of the change in the proportion of infant spit-ups for the LS (red dot plot) and BiLS (blue dot plot) groups during the Baseline, Intervention, and Post-intervention periods. n = 34 for each group during the Baseline and Intervention periods, and n = 33 for the LS and n = 34 for the BiLS groups during the Post-intervention period. (DOCX 51 kb) [file 12887_2017_886_MOESM9_ESM.docx]

**Figure S3** **Infant spit-ups.** Mean ± SD of the change in the proportion of infant spit-ups for the LS (red dot plot) and BiLS (blue dot plot) groups during the Baseline, Intervention, and Post-intervention periods. *n* = 34 for each group during the Baseline and Intervention periods, and *n* = 33 for the LS and *n* = 34 for the BiLS groups during the Post-intervention period.
