# Supplementary material for: Safety and tolerability of Bifidobacterium longum subspecies infantis EVC001 supplementation in healthy term breastfed infants: a phase I clinical trial
Source: BMC Pediatr. 2017 May 30;17:133. doi: 10.1186/s12887-017-0886-9 (PMC5450358; doi:10.1186/s12887-017-0886-9)
Supplement: Supplementary file 10 — Mean ± SD of the proportion of infant flatulence reported by mothers (red dot plot) and BiLS (blue dot plot) groups during the Baseline, Intervention, and Post-intervention periods. n = 17 for the LS and n = 12 for the BiLS groups for all time periods. (DOCX 54 kb) [file 12887_2017_886_MOESM10_ESM.docx]

**Figure S4 Infant flatulence.** Mean ± SD of the proportion of infant flatulence reported by mothers (red dot plot) and BiLS (blue dot plot) groups during the Baseline, Intervention, and Post-intervention periods. *n* = 17 for the LS and *n* = 12 for the BiLS groups for all time periods.
